# *In Vivo* Imaging of Microglia With Multiphoton Microscopy

**DOI:** 10.3389/fnagi.2018.00218

**Published:** 2018-07-19

**Authors:** Carmen Hierro-Bujalance, Brian J. Bacskai, Monica Garcia-Alloza

**Affiliations:** ^1^Division of Physiology, School of Medicine, Instituto de Investigación e Innovación en Ciencias Biomedicas de la Provincia de Cadiz (INiBICA), Universidad de Cádiz, Cádiz, Spain; ^2^Alzheimer Research Unit, Department of Neurology, Massachusetts General Hospital, Harvard Medical School, Harvard University, Boston, MA, United States

**Keywords:** multiphoton microscopy, Alzheimer’s disease, microglia, amyloid-beta, immunotherapy

## Abstract

Neuroimaging has become an unparalleled tool to understand the central nervous system (CNS) anatomy, physiology and neurological diseases. While an altered immune function and microglia hyperactivation are common neuropathological features for many CNS disorders and neurodegenerative diseases, direct assessment of the role of microglial cells remains a challenging task. Non-invasive neuroimaging techniques, including magnetic resonance imaging (MRI), positron emission tomography (PET) and single positron emission computed tomography (SPECT) are widely used for human clinical applications, and a variety of ligands are available to detect neuroinflammation. In animal models, intravital imaging has been largely used, and minimally invasive multiphoton microcopy (MPM) provides high resolution detection of single microglia cells, longitudinally, in living brain. In this study, we review *in vivo* real-time MPM approaches to assess microglia in preclinical studies, including individual cell responses in surveillance, support, protection and restoration of brain tissue integrity, synapse formation, homeostasis, as well as in different pathological situations. We focus on *in vivo* studies that assess the role of microglia in mouse models of Alzheimer’s disease (AD), analyzing microglial motility and recruitment, as well as the role of microglia in anti-amyloid-β treatment, as a key therapeutic approach to treat AD. Altogether, MPM provides a high contrast and high spatial resolution approach to follow microglia chronically *in vivo* in complex models, supporting MPM as a powerful tool for deep intravital tissue imaging.

## Neuroimaging Techniques: An Overview

In the last few decades there have been dramatic advances in clinical neuroimaging. These approaches serve as methods of diagnosis and prognosis, and provide the ability to monitor the natural history of patients and the progress of pharmacological treatments (Garcia-Alloza and Bacskai, 2004; Atri, 2016; Vegting et al., 2016). However, the techniques have also been adapted to follow chronically animal models of disease (Kang and McGavern, 2009) as well as to test new therapeutic alternatives. An ideal neuroimaging technique would provide spatial resolution to allow subcellular morphological and physiological studies, as well as high temporal resolution and sensitivity. Labeled ligands and tracers used should also be characterized to the extent that the metabolism of the compound does not interfere with the sensitivity or specificity of the ligand while being able to cross the blood-brain barrier. Also, the half-life of radioactive compounds must be long enough to allow their quantification and provide a high signal-to-noise ratio.

Among these techniques, positron emission tomography (PET) and single positron emission computed tomography (SPECT) are regularly used to allow three-dimensional, non-invasive imaging of the brain *in vivo*. Both PET and SPECT are very sensitive noninvasive *in vivo* imaging techniques, that allow the detection of neurotransmitters, neuroreceptors or transporters in the picomolar range. The spatial resolution for PET is relatively limited and by working with detector widths that balance spatial resolution and manufacturing limitations, the spatial resolution can reach ~1.0 mm for pre-clinical PET and ~3.0 mm for clinical PET (Moses, 2011). More typically, however, the spatial resolution of pre-clinical PET scanners is about 1–2 mm and about 4–6 mm for clinical PET scanners (Khalil et al., 2011; Table 1). PET tracers for inflammation have most recently focused on labeling the cannabinoid receptor type 2, cyclooxygenase-2, or reactive oxygen species (Janssen et al., 2018). However, the translocator protein (TSPO) 18 KDa, a mitochondrial molecule that gets upregulated when microglia is activated (Airas et al., 2018), is the most commonly used marker for microglia activation and inflammation in PET studies (Owen et al., 2017).

**Table 1:** *In vivo* neuroimaging techniques.

Technique	Preclinical spatial resolution	Clinical spatial resolution	Tracers/Labelers
Positron emission tomography (PET)	~1.0 mm	~3.0 mm	Radionuclei
Single positron emission computed tomography (SPECT)	<1 mm	8–12 mm	Radionuclei
Magnetic resonance imaging (MRI)	~10 μm	>100 μm	Magnetic nuclei
Intravital microscopy (IVM)	~10 μm	N.A.	Fluorophores
Multiphoton microscopy (MPM)	<1 μm	N.A.	Fluorophores

Preclinical SPECT resolution may also reach <1 mm and about 8–12 mm in the case of clinical SPECT (Khalil et al., 2011). In these cases, molecules labeled with positron emitting radionucleotides, such as [^15^O], [^11^C], [^18^F] have been widely used. As the radioactive isotopes decay, the resulting emission of detected γ-rays are used to image and measure biochemical processes *in vivo*. However, the short half-lives of [^15^O] or [^11^C] require the on-site presence of a cyclotron to produce the radioisotopes, limiting their widespread use, particularly in more rural areas. Techniques employing tomographic reconstruction are used to generate three-dimensional images and it is possible to measure metabolic processes, perform receptor-binding studies, and explore brain pathophysiology as well as drug treatment responses.

Spin properties of atomic nuclei with an odd number of protons or neutrons, such as [^1^H], [^13^C], or [^31^P], are used in magnetic resonance imaging (MRI). MRI usually refers to the representation of the spatial distribution of [^1^H] from water and fat molecules in the sample, allowing the anatomical identification of the areas under study as well as providing information about abnormalities in different pathologies (Dijkhuizen and Nicolay, 2003; Vargas et al., 2018). MRI resolution may reach ~10 μm in high magnetic field scanners (Jasanoff, 2007), but typical resolutions in clinical scanners are in the range of hundreds of microns (Table 1). Despite this advantage in spatial resolution, MRI is much less sensitive than PET or SPECT. The sensitivity requires high concentrations of the imaged molecule, in the high micromolar to low millimolar range, to be detected (Caravan, 2009). The administration of exogenous contrast media allows the measurement of biological processes in some occasions, although the applications are limited.

Classical intravital microscopy (IVM) has long evolved in the last decades and provides microscopic resolution to follow and analyze physiological and pathological processes in live animals (Pittet and Weissleder, 2011). Confocal IVM can follow cellular events up to 200 μm deep, however resolution is compromised at deeper depths due to of out-of-focus emission light and scattering of in-focus emission light (Taqueti and Jaffer, 2013), with general resolution in the tens of microns. This approach has been largely used to assess cellular processes including cell development, migration or death (Pittet and Weissleder, 2011), vascular events and immune system responses, (Aulakh, 2018; De Giovanni and Iannacone, 2018; Russo et al., 2018), including microglia (Bayerl et al., 2016), among others. Recent approaches have allowed the application of IVM to cancer diagnosis and tumor vessel characterization in patients (Gabriel et al., 2018). Also, novel advances make it possible to work with more fluorescent channels, longer imaging times and larger depths in animal models (but still limited to <250 um; for review see Pittet and Weissleder, 2011). While extremely useful in animal studies, it has been the development of multiphoton microscopy (MPM) that has significantly improved the possibility of deep (upto 1 mm), chronic *in vivo* imaging at the subcellular level.

MPM offers very high spatial resolution, in the range of micrometers, and very fast imaging acquisition. Optical imaging precludes the need for radioactive ligands used in PET and SPECT and the large number of fluorescent ligands allows extremely diverse structural and functional readouts. The main disadvantages are that the approach is invasive and the fact that only a limited portion of the brain can be assessed, restricting its use to animal imaging. Nevertheless, this is a very powerful approach for animal studies. MPM has been largely used as a reference technique to explore the central nervous system (CNS) morphology and function in preclinical studies that include neural network activity, synaptic development, brain damage, immune system responses and the role of microglia, progressive pathology or cellular responses in different pathological situations.

## Principles and Advantages of Multiphoton Microscopy

MPM is based on the probability that two or more low energy photons interact nearly simultaneously with a fluorescent molecule. This induces an electronic transition comparable to the absorption of one photon with double the energy. Then, a single photon is emitted by the excited fluorophore (Denk et al., 1990). By reaching <1 μm spatial resolution, MPM allows cellular and subcellular discrimination without suffering from the slow image acquisition of MRI and PET.

MPM offers advantages over other modes of fluorescence or confocal fluorescence, that have been previously reviewed (Oheim et al., 2006; Svoboda and Yasuda, 2006). Briefly, MPM uses low energy, near infrared light, with wavelengths above 700 nm, reducing phototoxicity and tissue damage, as excitation is limited to the plane of focus. This allows chronic *in vivo* imaging over long periods, without significantly damaging imaged areas. Absorption and scattering are limited when compared to UV or visible light, so excitation penetrates deeper into the sample, and the loss that occurs can be compensated, at least partially, by optimizing signal collection with efficient photomultiplier tubes. Since MPM fluorescence is limited to the point of focus of the objective, out of focus fluorescence is greatly reduced. Common MPM imaging depths reach ~500 μm and different approaches have been developed to gain even deeper access (~1 mm). Additionally, gradient index lenses allow the possibility of acquiring images of high quality a few centimeters from the object plane, with modest tissue damage (Levene et al., 2004; Velasco and Levene, 2014; Moretti et al., 2016). However, the surrounding sites are likely to be damaged, making this approach much more invasive. Another possibility to further increase the depth of imaging in highly scattering environments, such as brain tissue, is the use of longer wavelengths. By using a spectral excitation window of 1700 nm, subcortical structures within an intact mouse brain can be reached (Horton et al., 2013). These approaches open the door to image deeper structures of the mouse brain, including the corpus callosum or even the hippocampus. Also, other dense structures such as the kidney, skin or muscle might be imaged (Miller et al., 2017). Additionally, the multiphoton absorption spectrum is much broader than the single photon absorption spectrum. Multiphoton excitation at a single wavelength stimulates multiple fluorophores without requiring multiple lasers or other illumination sources required for multi-color fluorescence imaging. Therefore, simultaneous assessment of different probes is feasible due to the large variety of fluorophores available that allows true multiplexing. In order to fully explore this option, sophisticated fluorescent sensors to monitor oxidative stress (Xie et al., 2013; Wagener et al., 2016; Galvan et al., 2017), oxygen distribution (Gagnon et al., 2016), calcium homeostasis (Kuchibhotla et al., 2014; Eikermann-Haerter et al., 2015; Arbel-Ornath et al., 2017; Bai et al., 2017), chloride concentration assayed by Clomeleon imaging (Dzhala et al., 2010) or potassium sensors (Sui et al., 2015) have been developed to observe cellular and subcellular structures and activity *in vivo*. Moreover, cells also produce autofluorophores, such as porphyrins, NAD(P)H, flavin, lipofuscin, melanin or collagen, allowing the imaging of cellular and metabolic processes using intrinsic signals. Therefore, MPM has revealed unanticipated new principles and mechanisms after imaging and reimaging at both acute and chronic time points (Akassoglou et al., 2017). Additionally, the fact that MPM provides high contrast and high spatial resolution suggests that it has promise as a tool for specialized intravital deep tissue imaging in certain clinical studies (Wang et al., 2010).

## Technical Approaches for Brain *in Vivo* Multiphoton Imaging

MPM has been used to study many biomedical problems, including applications in urology (Katz et al., 2014), tumor assessment (Muensterer et al., 2017), infectious diseases (Belperron et al., 2018) and cardiovascular research (Wu et al., 2017), among others. However, MPM has been most widely used in neuroscience studies for pathophysiological assessment and follow-up of the brain. Insights of the CNS provided by MPM were not previously possible with classical histological endpoint studies. Minimally invasive MPM provides high spatial resolution for imaging glial cells, vascular structures and single neurons, as well as spines and subcellular components in the intact brain. Moreover, MPM allows functional measures of brain physiological processes, as well as dynamic responses to injury and disease in real time (Akassoglou et al., 2017).

**Figure 1:**
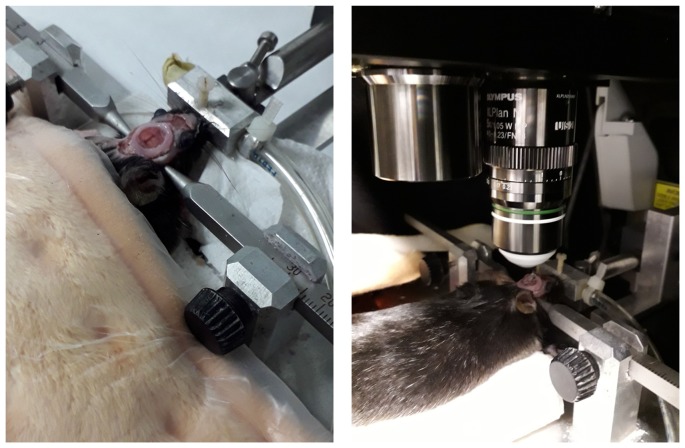
Representative images of cranial window implantation and imaging setup for real time *in vivo* brain imaging with minimally invasive multiphoton microcopy (MPM).

A craniotomy is required to directly access the brain. The so-called “open skull” technique requires the permanent removal of a circular portion of skull, with or without altering the dura, and the placement of a cover glass (Arbel-Ornath et al., 2017; Askoxylakis et al., 2017; Figure 1). While this is the most common approach, it is also possible to image the brain through a “thin skull” preparation (Marker et al., 2010). In this case, the skull of living animals is thinned down to ~15 μm (Christie et al., 2001; Allegra Mascaro et al., 2010; Isshiki and Okabe, 2014). This approach is slightly more challenging technically, but it does not compromise intracranial pressure, even in the short term. Whereas the open skull window technique is more invasive, it also allows higher flexibility, with larger imaged areas of the brain and longer imaging periods (Isshiki and Okabe, 2014). However, while the resolution of both cranial window preparations is comparable in the superficial layer of the neocortex, it has been reported that at points deeper than 50 μm, the thin skull windows suffer from lower imaging quality (Isshiki and Okabe, 2014), making open skull more appropriate when nanosurgery approaches, such as neuronal ablation or individual blood vessel disruption (Allegra Mascaro et al., 2010; Garcia-Alloza et al., 2011) are required. Also, open cranial windows allow larger fields of view, that can be chronically imaged in the long term, while thin skull preparations require bone thinning at every experimental session. Repeated skull thinning may induce variations in imaging quality between sessions, limiting the number of successful reimaging sessions (Holtmaat et al., 2009).

As an evolving technique, other types of windows have been developed in the last few years to accommodate new experimental necessities, such as cerebellar implantation and imaging (Askoxylakis et al., 2017). Also, transparent, silicone-based polydimethylsiloxane membranes have been used as coverslips. These membranes are not only transparent but they allow access to the brain with microelectrodes (Heo et al., 2016), so electrophysiology studies can be performed simultaneously. Also, removable and replaceable windows have been developed for chronic widefield imaging in awake head-fixed mice (Goldey et al., 2014), making this technique particularly useful for the study of chronic diseases or local pharmacological treatments. Both thin skull and open window preparations have been carefully reviewed before (Allegra Mascaro et al., 2010; Holtmaat et al., 2009; Mostany and Portera-Cailliau, 2008), and new technical approaches have been described to overcome some of the difficulties associated with the techniques (Goldey et al., 2014; Heo et al., 2016).

### Cranial Window Implantation

Cranial window surgeries have been performed with slightly different approaches and detailed protocols have been previously described (Mostany and Portera-Cailliau, 2008). Briefly, before the commencement of the procedure, the stereotaxic frame and all surgical surfaces are disinfected. Surgical material including forceps, scissors, drill and glass coverslips are sterilized in a micro bead sterilizer. Small pieces (~1.5 mm^2^) of gel foam are soaked in sterile saline to be used during the surgical procedure. Animals are anesthetized with isoflurane: ~3% for induction, in an induction chamber, and ~1% for the surgery while continuously monitoring animal reflexes. Body temperature is maintained with a water recirculating blanket during the entire surgery-imaging procedure, and until the animal is fully awake. To begin the surgical procedure, the sedated animal is placed in a stereotaxic frame and eye ointment is applied to prevent dry eyes. The hair is trimmed in between the eyes from the neck to the eyes and the area is swapped with cotton tips dipped in iodopovidone and ethanol alternatively, for three times, to disinfect the skin surface. Local anesthesia (lidocaine) is subcutaneously injected and anti-inflammatory drugs (corticoids or non-steroid anti-inflammatory drugs) can also be administered before commencing the surgery. With a scalpel and scissors the skin is opened and the muscles and periosteum are carefully removed to guarantee access to the cranium. Initial drilling is performed to mark the cranium area between Bregma and Lambda to be removed (~6 mm in diameter). The skull is thinned in a circular pattern by careful drilling until it is almost detached. At this moment the area is removed with fine forceps and wet gel foam is gently applied to the brain surface to limit swelling and bleeding. If dura matter needs to be removed, fine forceps can be used to pull it gently from the craniotomy borders and leave it on the midline without damaging the leptomeningeal vessels. The coverslip (8 mm in diameter) is dipped in sterile saline, placed on top of the craniotomy and attached to the cranium with dental cement. Before finishing the procedure, the animal receives subcutaneous opioids (buprenorphine), and acetaminophen is administered in the drinking water for the next three consecutive days. Animals can then be imaged acutely, or allowed recover from the surgery for several days or weeks. A well-performed surgery leads to windows that allow longitudinal imaging for up to 1 year or even longer periods.

## Approaches for *in Vivo* Multiphoton Microglia Imaging in Physiological and Pathological Conditions

Microglia constitute ~5%–12% of all cells in the mouse brain, and while their density varies among brain regions, they are prevalent in the gray matter (Brawek and Garaschuk, 2017). At the simplest level, microglia are the immune cells of the brain. They have a distinct developmental origin that differentiates them from other myeloid cells in the CNS (Tvrdik and Kalani, 2017). Microglia play relevant roles in the support, surveillance, protection and restoration of tissue damaged in the CNS (Davalos et al., 2005; Neumann et al., 2009; Schafer et al., 2012). However, microglia have also been implicated in neurogenesis and synapse formation and pruning (Akerblom et al., 2013), giving them a much more complex role in brain homeostasis and function.

Immunolabeling microglia in *ex vivo* or *postmortem* brain tissues provides only a snapshot of complex dynamic processes, while *in vivo* imaging of microglia allows the study of these cells in their environment over time and their implication in physiological and pathological situations of the CNS (Venneti et al., 2009). Multiple and complex techniques have been developed such as confocal microscopy in zebra fish embryos, PET in larger animal models and humans, or multiphoton imaging (Venneti et al., 2009). In the latter approach, the development of fluorescent engineered probes and mice has allowed chronic *in vivo* monitoring not only of synaptic, dendritic and neuronal alterations (Li and Murphy, 2008; Garcia-Alloza et al., 2011; Bai et al., 2017) or brain vascular events (O’Herron et al., 2016; Taylor et al., 2016), but also glial activity (Galea et al., 2015; Füger et al., 2017; Lind et al., 2018; Stobart et al., 2018).

While *in vivo* imaging is technically challenging to visualize and follow microglia, many efforts have been directed towards faithfully monitoring these cells by MPM in real time. Some approaches to visualize microglia have included the use of commercially available plant lectins conjugated with fluorophores, such as conjugated Tomato lectin or Isolectin IB4 (Brawek and Garaschuk, 2017). However, labeled volumes are limited, endothelial cells surrounding blood vessels are also labeled and the quality of labeling depends on the use of the specific plant lectin. Also, a major limitation is that longitudinal imaging is not possible since the requirement for intraparenchymal injection of the fluorophore limits the approach to acute experiments (Brawek and Garaschuk, 2017). To overcome this restriction, the delivery of viral vectors, inducing stable expression of fluorescent proteins in microglia have been used, such as microRNA-9-regulated vectors. Degradation of the transgene messenger RNA in microglia is induced by the incorporation of complementary microRNA-9 target sites into the transgene cassette (Brawek and Garaschuk, 2017; Table 2). Since rodent microglia lack miR-9 expression, microRNA-9-regulated expression of GFP in the brain parenchyma can be used to label these cells. However, NeuN colabeling has also shown the existence of neurons, without miR-9 activity, that express GFP (Akerblom et al., 2013), indicating that miR-9 activity is not entirely selective. Also, the fact that the strength of the GFP expression varies depending on the microRNA-9 activity may limit the applicability of this tool in disease models. On the other hand, recombinant adeno-associated viruses (rAAV) have also been tested to express GFP in microglial cells (Table 2). Multiple rAAV have led to efficient gene expression from neurons, oligodendrocytes, and astrocytes when injected into the brain, however rAAV-mediated genetic targeting of cells of myeloid lineage, and specifically microglia, remains challenging. Recent approaches with capsid-modified adeno-associated virus six vectors and microglia-specific promoters (scF4/80 and scCD8) have reported selective GFP expression in primary mixed neuroglial cultures and *in vivo* after intracerebroventricular injections in wildtype mice. However, since aberrant activation of microglia may induce pathological conditions, manipulating microglia function could result in disease modification in such intractable diseases (Rosario et al., 2016).

**Table 2:** Multiphoton *in vivo* tools for microglia imaging.

Labeling approach	Options for multiphoton *in vivo* imaging
Histochemical labeling	Plant lectin conjugated with fluorophores (tomato lectin, isolectin IB4) (Brawek and Garaschuk, 2017)
Genetic labeling	Knock in CX3CR1-GFP mice (Jung et al., 2000) CX3CR1-EGFP mice (Nimmerjahn et al., 2005) CD11b-CreERT2;R26-tdTomato mice mice (Füger et al., 2017) CD11b-CreERT2;R26-tdTomato; Iba1-eGFP mice (Füger et al., 2017)
Viral labeling	Viral vectors: microRNA-9-regulated vector (Brawek and Garaschuk, 2017) Recombinant adeno-associated viruses (rAAV) (Rosario et al., 2016)
Calcium indicators	Oregon Green BAPTA-1 GCaMP2 (retroviral vector) (Seifert et al., 2011) Twitch-2B (lentiviral vector) (Brawek et al., 2017) GCaMP5G mice (genetically encoded) (Gee et al., 2014).

Even though some limitations still apply, microglial expression of the chemokine fractalkine receptor, CX3CR, has allowed the generation of knockin CX3CR1-GFP (Jung et al., 2000) or -EGFP (Nimmerjahn et al., 2005) mice and conditional models (Parkhurst et al., 2013; Yona et al., 2013) as the most widely used approach to visualize microglia *in vivo* and in real time. The microglia are brightly labeled in these mice, allowing *in vivo* visualization of the microglial cell morphology, including very fine processes (Brawek and Garaschuk, 2017), allowing the assessment of microglia morphology and function *in vivo* (Bennett et al., 2016). While extremely useful, it is important to bear in mind that in these mice the CX3CR1 gene is replaced by GFP, generating a partially functional KO of the receptor. Therefore, these mice can be used to study the receptor function, however, the partial CX3CR1 KO might also induce functional alterations (Brawek and Garaschuk, 2017).

### Imaging Microglia With Multiphoton Microscopy in Physiological Conditions

CX3CR1-EGFP mice have allowed the characterization of cortical microglia using MPM (Nimmerjahn et al., 2005; Table 2). Microglia are characterized by a ramified morphology in a resting state. *In vivo* multiphoton imaging studies have revealed that even in this resting state, microglia show filopodia-like protrusions that are highly motile and of variable shape (Nimmerjahn et al., 2005). Microglia also radially extend and retract their processes at an average rate of 2.2 ± 0.2 μm/min, to survey and assess their environment, while they continuously interact with other surrounding elements (Nimmerjahn et al., 2005). A recent study described fundamental concepts of microglial function and lifespan directly, by *in vivo* MPM imaging (Füger et al., 2017). Alternative approaches to label individual microglia include triple-transgenic mice that also carried an Iba1-eGFP transgene or double-transgenic CD11b-CreERT2;R26-tdTomato mice (Table 2). Microglia were followed chronically for 15.5 months in young (3 months old) and old (10 months old) mice. Median microglial lifetime span was estimated to be 29 months in young mice and 22 months is older animals, matching approximately the mean age difference of the two groups of mice imaged. These data support that microglial proliferation in the mouse neocortex appears to be a rather deactivated process and that about half of the microglia persist until the end of the 26–28-month mean lifespan of C57BL/6 mice. This extreme longevity may explain how stimulation of microglia early in life might be crucial for long-term changes in human brain function and the risk of neurodegenerative diseases (Füger et al., 2017). Further assessment of microglial proliferation in young animals revealed that the number of cells that were lost or appeared was similar over the 6-month period when mice were imaged biweekly. Since new tdTomato-expressing cells were reported to appear in the proximity of an existing tdTomato-expressing microglial cell after an increase in cell body volume, Füger et al. (2017) speculate that these are the mother cells resulting in the newly generated microglia. Newly generated microglia cells then move away and extend their ramified processes interconnecting with the existing microglial networks (Füger et al., 2017). On the other hand, previous *in vivo* multiphoton studies have also shown that a small population of circulating CX3CR1-GFP cells may infiltrate the brain parenchyma through a compromised blood-brain barrier, induced by ischemic stroke. It has been reported that these cells do not proliferate and are morphologically distinguishable from resident microglia, suggesting that infiltration and proliferation are two independent events, with different kinetics (Li et al., 2013). On the other hand, chronic multiphoton *in vivo* studies in CX3CR1-GFP have reported that microglia are fast and continuously remodeling. Askew et al. (2017) show that the number of microglia is maintained until aging due to temporal and spatial coupling of proliferation and apoptosis, due to the fact that many more cells proliferate in the close proximity of a dying cell.

### Imaging Microglia With Multiphoton Microscopy in Pathological Conditions

**Figure 2:**
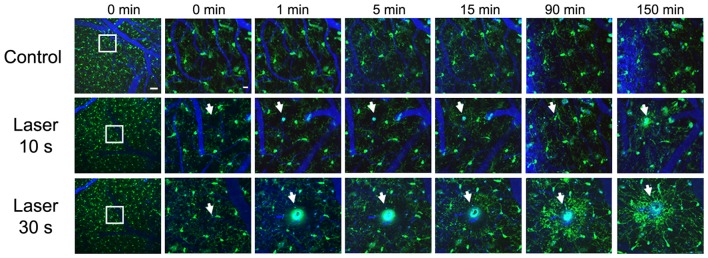
Real time *in vivo* multiphoton imaging of microglia in the cerebral cortex from CX3CR1-GFP mice. Representative images of sequential follow up of a control mouse and two mice after parenchyma laser ablation for 10 and 30 s. Same regions are imaged before the lesion, immediately after, 1, 5, 15, 90 and 150 min afterwards. Microglia processes start going towards the lesion site as soon as 1 min after laser ablation and the processes continue extending towards the lesion 150 min later. Vessels (blue) are filled with Texas Red dextran 70 KD, microglia (GFP, green) and white arrows point to laser ablation sites. Scale bar 50 μm and insets 12 μm.

While the concept of resting and activated microglia might be ambiguous, since different phenotypic and morphological stages of microglia are represented *in vivo* (Madore et al., 2013), previous studies have analyzed the acute effect of different lesions on microglial responses. *In vivo* multiphoton imaging studies have revealed that microglia extend their processes towards an injured area, depending on the severity of the lesion, in an ATP-dependent fashion (Davalos et al., 2005). Figure 2 shows real time *in vivo* imaging of how parenchymal laser ablation triggers microglia processes towards the lesion area in CX3CR1-GFP mice, and this effect seems to depend on the severity of the lesion. Likewise, laser disruption of blood-brain barrier induces a prompt microglia response, that includes a change from undirected to directed movement of nearby microglial processes toward the injured area (Nimmerjahn et al., 2005). Also, in response to an injury, such as an ischemic stroke, Aβ deposition (Lull and Block, 2010) or probe implantation (Kozai et al., 2012), it has been reported that microglia change into an ameboid morphology and activate several markers such as CD68 and MHCII (Kettenmann et al., 2011; Lartey et al., 2014). Specifically, after electrode implantation, microglial cells adjacent to the probe focus their processes toward the probe while cells distant to the probe maintain ramified morphology. Moreover, microglia cell body displacement toward the probe is also detected (Kozai et al., 2012).

Once microglia are activated, an immune cascade begins, including the production of many cytokines, chemokines and reactive oxygen and nitrogen species as well as phagocytosis of dead cells (Dheen et al., 2007; Jin et al., 2010). The role of microglia in synaptic function has also been analyzed by MPM *in vivo*, and previous studies have shown that the physiological and pathological state of the local brain environment determinates the associated response of microglial processes and synapses (Wake et al., 2009). It appears that microglial processes and neuronal synapses make intimate but transient connections, that are dependant on the functional status of the synapse. In this sense, it has been suggested that microglia may contribute to the increased turnover of synaptic connections observed in injured areas after long-lasting microglial contact, (Wake et al., 2009). On the other hand, recent studies, focusing on the close relationship between microglia and synapses, have suggested that the role of microglia at this level might be more complex than mere phagocytosis. Rapid microglial trogocytosys of presynaptic components is observed, as well as the induction of postsynaptic spine head filopodia, suggesting a facilitator role of microglia in synaptic remodeling and maturation (Weinhard et al., 2018). It has also been reported that selective microglia elimination deregulates neuronal network activity after stroke (Szalay et al., 2016).

Other multiphoton *in vivo* approaches have been directed to study the role of microglia in multiple sclerosis. In an animal model, Davalos et al. (2012) have shown that microglia movement is triggered by the plasma protein fibrinogen before neurological signs of multiple sclerosis commence in the spinal cord, and that microglia clusters around vessels facilitate local axonal damage as the disease progresses. Following these approaches, other *in vivo* studies have focused on exploring therapeutic agents that lower microglial activation and hence neuroinflammation (Bok et al., 2015). Also, real time *in vivo* microglia assessment has been used to further study other CNS diseases such as glioblastoma multiforme models, where activated microglia were found to contact glioma cells shortly after tumor seeding (Resende et al., 2016).

As described above, some studies have assessed microglia structure and motility *in vivo* with real time imaging, and since microglia are non-excitable cells, they depend on changes in the intracellular concentration of calcium to communicate with other cells. However, calcium indicators for microglia have faced some challenges when used *in vivo* (Brawek and Garaschuk, 2017). First approaches have used single-cell electroporation of individual cortical microglial cells with the calcium indicator Oregon Green BAPTA-1 to analyze calcium signals. While the majority of microglial cells presented no spontaneous calcium transients at rest and during strong neuronal activity, they respond with large, generalized calcium transients to an individual damaged neuron (Eichhoff et al., 2011). Alternative approaches have included the use of retroviruses encoding the calcium sensor GCaMP2, in the cortex. However, since retroviruses only infect dividing cells, to stimulate microglial proliferation, the authors used stab wound injury (Seifert et al., 2011). Other studies have included the development of a mouse line that expresses the single-wavelength calcium indicator, GCaMP5G, and the red fluorescent protein tdTomato (Gee et al., 2014). This reporter shows strong expression in different cell types, including microglia and astrocytes (Tvrdik and Kalani, 2017), showing that calcium activity decreases at later stages of inflammation, especially after microglia acquire ameboid morphology in response to LPS challenge. Likewise, calcium responses to laser lesions remain low for at least 1 month after a single LPS administration (Tvrdik and Kalani, 2017). Recently Brawek et al. (2017) have also reported the use of lentiviral vectors driving expression of the calcium reporter Twitch-2B in microglia as a feasible approach to analyze calcium signaling with some selectivity in cortical microglial cells. All of these promising tools will help to evaluate microglial immunomodulation in physiological and pathological brain conditions.

## *In Vivo* Imaging of Microglia in Alzheimer’s Disease Models

Senile plaques, neurofibrillary tangles and synaptic and neuronal loss are the classical neuropathological features of Alzheimer’s disease (AD; Serrano-Pozo et al., 2011). Also, microglia play a significant part in CNS diseases and specifically in AD. Microglia have a dual role in AD and it has been described that they can be protective and promote phagocytosis, degradation and ultimately clearance of amyloid-β (Aβ) with disease progression. On the other hand, microglia become dysfunctional, their ability to clear Aβ is affected and they release neurotoxins and produce pro-inflammatory cytokines that contribute to Aβ production and deposition (Hickman and El Khoury, 2014). Altogether, it seems that the microglia role in clearing Aβ is compromised while their inflammatory activity in AD is increased (Sarlus and Heneka, 2017). Microglia clusters around senile plaques are observed in AD patients and animal models, and it has been suggested that the brain can compensate for Aβ toxic effects of up to a limited level (Baron et al., 2007) in which microglia may play a relevant role. On the other hand, microglia impairment seems to increase amyloid burden (El Khoury et al., 2007) and alter plaque structure with significantly greater neuritic damage (Wang et al., 2016). Also, the positive role of microglial activation in removing Aβ can be compromised by the concomitant effect of an increased secretion of proinflammatory compounds that might be toxic for nearby neurons.

In the last few years, a rare functional variant (R47H) in triggering receptor expressed on myeloid cells (TREM) two gene, encoding TREM2 protein, has been reported to increase susceptibility to late-onset AD through impaired containment of the inflammatory processes (Guerreiro et al., 2013; Jiang et al., 2013; Jonsson et al., 2013). TREM2 can regulate the inflammatory response of myeloid cells and their phagocytic ability. It has also been reported that TREM2 expression is positively correlated with amyloid deposition in individuals with AD and upregulated around plaques in AD models. Furthermore, an overall decrease in microgliosis surrounding Aβ plaques is observed in TREM2 haploinsufficient and TREM2 deficient mice (Ulrich and Holtzman, 2016). TREM2 enables microglia to circumscribe and interfere with Aβ plaque structure, limiting neuronal damage and ultimately protecting from AD (Wang et al., 2016). In this context, microglia accumulation is likely neuroprotective, helping to contain the spread of plaques and shield the rest of the brain from the synaptotoxic Aβ oligomers (Hong and Stevens, 2017). However, in TREM2 deficient mice Aβ plaques are not fully enclosed by microglia and are associated with significantly greater neuritic damage (Wang et al., 2016). Additional efforts are required to define the role of TREM2 in health and disease.

Moreover, microglia have been implicated in synaptic pruning in the developing brain and it still needs to be determined whether normal pruning could be activated and mediate synaptic loss in the AD brain before senile plaque deposition commences. In this sense, microglia engulf synaptic elements, that are internalized into lysosomal compartments in a manner similar to developmental synaptic pruning, when challenged with oligomeric Aβ (Hong et al., 2016). However oligomeric Aβ does not increase synaptic engulfment in microglia lacking complement receptor 3 (CR3), showing that CR3 is necessary for oligomeric Aβ-dependent engulfment of synapses by microglia (Hong et al., 2016). Therefore, the therapeutic objective would be to reduce the neurodegenerative phenotypes of microglia, implicated in secreting pro-inflammatory cytokines, without affecting the beneficial role of microglia implicated in amyloid clearance. Despite the relevant role that microglia play in AD, only a handful of studies have analyzed these cells using *in vivo* MPM. Most of this work has focused on the role that microglia plays in reducing or exacerbating amyloid pathology (Meyer-Luehmann et al., 2008; Venneti et al., 2009) or the implication of microglial cells in the clearing process after anti-Aβ treatments (Koenigsknecht-Talboo et al., 2008; Garcia-Alloza et al., 2013).

### *In Vivo* Study of Microglia Dynamics in AD Models

**Figure 3:**
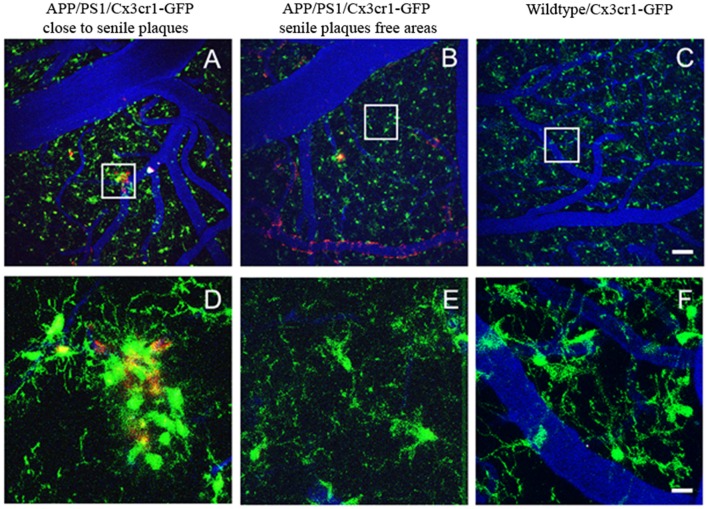
Real time *in vivo* multiphoton imaging of microglia in the cerebral cortex from APP/PS1×CX3CR1-GFP mice. Representative images of microglia clusters around senile plaques **(A,D)**, in areas free from senile plaques **(B,E)**, and in a wildtype mouse **(C,F)**. Vessels (blue) are filled with Texas Red dextran 70 KD, senile plaques are labeled with methoxy-XO4 (red), and microglia express GFP (green). The scale bar is 50 μm and for the insets 10 μm.

While it has been reported that activated microglia accumulate Aβ in lysosomes, preceding neuronal death (Baik et al., 2016) and secondarily contributing to plaque formation, the majority of the *in vivo* multiphoton studies support that microglia recruitment follows plaque formation, and not the other way around (Bolmont et al., 2008; Meyer-Luehmann et al., 2008). Initial studies in AD mice (PDAPP) crossed with CX3CR1-EGFP mice (PDAPPxCX3CR1-EGFP) provided for the first-time the temporal relationship between amyloid deposits, microglial recruitment and the time-course of activation *in vivo* (Meyer-Luehmann et al., 2008). PDAPPxCX3CR1-EGFP mice were imaged before and after plaque formation. The study described in detail how senile plaques deposit in hours, and that microglia subsequently cluster around these newly formed deposits. Microglia were detected at the site of plaque formation within 1–2 days of a new plaque’s appearance, and while existing microglia remained stable, new microglia were dynamically recruited to the new plaques. On the other hand, none of the new plaques appeared immediately close to resident microglia, suggesting that microglia do not provide the nidus to form a new plaque. It was also implied that microglia do not successfully clear plaques, unless further activated, suggesting that they restrict senile plaque growth instead, and contribute to the steady state of plaque size after initial formation (Meyer-Luehmann et al., 2008). Other studies have shown similar results, and chronic *in vivo* imaging of microglia clusters did not predict the deposition of new senile plaques (Garcia-Alloza et al., 2013), as can be observed in Figure 3. Comparable observations have also been reported in other AD models (APP/PS1×Iba-1-GFP mice) after long-term *in vivo* microglia imaging (Bolmont et al., 2008). In depth analysis of microglia dynamics showed that the number of microglia increases over a month, independent of the volume of senile plaques. While larger plaques were surrounded by larger microglia, the average microglia size appeared to be stable over time.

Although individual microglia surrounding amyloid plaques remained stable for a month, new microglia were also observed. It was also shown that resting microglia first send processes and then migrate to contact a plaque (Bolmont et al., 2008). Likewise, Hefendehl et al. (2011) showed a local increase in the number of microglia cells surrounding amyloid plaques in APP/PS1×Iba1-GFP mice after long-term *in vivo* imaging, for up to 25 weeks. These authors also reported a limited role of the imaging or surgery processes on microglia morphology or number, whereas a relevant effect of pathology-related changes were reported, as previously described (Bolmont et al., 2008).

Surprisingly, it has been shown that laser ablation in 5XFAD/CX3CR1-GFP mice may induce methoxy-X04-positive deposits 2 weeks later in the damaged area, suggesting that microglial activation by laser irradiation promotes plaque formation *in vivo* (Baik et al., 2016). Mixed AD models have also been used to assess neuron-microglia interaction (Fuhrmann et al., 2010). The observed neuronal loss in layer III from APP/PS1/tau/CX3CR1-GFP/YFP-H was rescued by knockout of the chemokine receptor CX3CR1, suggesting that microglia are necessary for neuron elimination. The density of microglia was significantly increased over time around the lost neurons, within a range of 100 μm, and the total turnover rate of fine processes in individual microglial cells was reduced. It appears that microglia are recruited to the neuron with increased migration velocity before neuronal elimination. The authors suggest that this finding might result from the increased number of microglia around disappearing neurons in APP/PS1/tau/CX3CR1-GFP/YFP-H mice, indicating that maintenance of brain tissue is carried out by other cells (Fuhrmann et al., 2010). Since CX3CR1 knockouts did not change the levels of Aβ in the transgenic mouse models of AD, it appears that the phagocytic activity of microglia was not altered or was not involved. Additionally, the *in vivo* follow up of microglia in PDAPPxCX3CR1-GFP, before (3.5 months old) and after (14–17 months old) amyloid deposition demonstrated that young mice extend and retract their processes significantly more than microglial cells in older mice. The microglial cells in the immediate vicinity of amyloid pathology in older mice, as well as in areas distant from pathology, were stable and showed significantly less process movement when compared to young mice (Koenigsknecht-Talboo et al., 2008). These observations are in agreement with previous studies showing that the expression of receptors and enzymes involved in Aβ removal by microglial cells is progressively downregulated in AD mice (Hickman et al., 2008), supporting the idea that microglia at later stages of AD become dysfunctional and less efficient at removing and degrading Aβ (Hickman et al., 2008; Wake et al., 2009).

Recently, Füger et al. (2017) have also followed the natural history of microglia in an AD mouse model (CD11b-CreERT2;R26-tdTomato;APPPS1 mice). These authors reported a higher microglia cell loss in AD mice (~20%) when compared with wildtype mice (~13%) at 10 months of age. They also showed that cell division of non-plaque-associated microglia was over three times more common than microglia loss in APP/PS1 animals. Moreover, in the APP/PS1 mice, newly formed microglia were reported to move toward nearby amyloid plaques. Since the rates of plaque-associated microglia disappearance and proliferation were similar, the authors postulated that the increase in the numbers of microglia surrounding plaques, results from the proliferation of microglia in plaque-free areas. However, it cannot be ruled out that plaque-associated tdTomato-positive cells are derived, at least partially, from peripheral myeloid cells. Altogether, it seems that microglial motility and recruitment, assessed by multiphoton imaging, are affected in different ways, with results depending on specific amyloid depositing transgenic mouse models.

### *In Vivo* Microglia Response to Aβ Immunotherapy

Anti-Aβ immunotherapy triggered an increased interest in the role of microglia as the mediators of neuroinflammation in the AD brain. In this regard, a comprehensive analysis of the underlying mechanisms of antibody-mediated clearance and microglia activation could improve immunotherapy treatments for AD, while avoiding negative inflammatory side effects (Garcia-Alloza et al., 2013). The direct effect of anti-Aβ antibodies on amyloid deposition and clearance has been followed in detail *in vivo* and in real time with MPM (Bacskai et al., 2002; Prada et al., 2007). It has been reported that direct antibody administration increases the microglial response (Krabbe et al., 2013). However, it seems that activating or inhibiting microglia *per se* has a limited role in eliminating senile plaques (Garcia-Alloza et al., 2007). Additionally, *in vivo* studies using Fab2 fragments of the anti-Aβ 3D6 antibody, lacking the microglial activating Fc portion, demonstrated that these are as effective in eliminating Aβ as complete antibodies, indicating that clearance of amyloid deposits *in vivo* may involve a non-Fc-mediated disruption of plaque structure (Bacskai et al., 2002). While acute peripheral administration of 3D6 antibody does not seem to affect microglia dynamics in PDAPPxCX3CR1-GFP mice, antibody treated mice presented significantly more cells, with twice as many processes (Koenigsknecht-Talboo et al., 2008) and this effect was particularly striking around senile plaques. However, the effect seems to be limited to older PDAPP mice (14–17 months of age), while no differences were observed in younger mice (3.5 months old), suggesting that the microglial response is only detected when aggregated Aβ is present, at later stages of the disease.

Other studies have also reported that long-term peripheral administration of anti-Aβ treatment not only reduces senile plaque load, but also restores microglial phagocytic capacity in APP/PS1 mice in the hippocampus, suggesting that restoring microglia activity might provide an attractive therapeutic approach even at advanced stages of AD (Krabbe et al., 2013). It has also been shown that direct administration of anti-Aβ antibodies to APP/PS1/CX3CR1-GFP mice increases microglia size and the number of processes in the close proximity to senile plaques within 1 week. A tendency towards increasing the numbers of cells located in the immediate surround of the senile plaques was also observed (Garcia-Alloza et al., 2013). The fact that senile plaques were not cleared in untreated mice, even though extensive microglia numbers were detected around amyloid deposits, supports the idea that mechanisms, both dependent and independent of microglia, may act in the immunotherapy mediated clearance of Aβ plaque (Garcia-Alloza et al., 2013). Together, it seems that senile plaques are a triggering factor to form microglia clusters and support the idea that while microglia do not seem to successfully clear plaques by themselves, they might be activated by anti-Aβ antibodies and contribute to Aβ clearance. However, the underlying mechanisms remain to be completely elucidated.

## Potential Caveats of *in Vivo* Imaging of Microglia

Chronic real time *in vivo* microglia imaging provides a powerful tool to help untangle the role of microglia in physiological homeostasis and in neurodegenerative diseases, specifically in AD. With this idea in mind, engineered animal models have been developed to follow morphological changes and dynamics of microglia. Even though functional imaging data are highly desirable, these tools are still limited (Tvrdik and Kalani, 2017). On the other hand, taking into account the critical role of microglia in neuroinflammation, the surgical approach for multiphoton *in vivo* imaging may induce microglia activation itself, and this caveat must be acknowledged. However, previous studies have reported a very limited effect of the surgeries in the performance of microglia (Garcia-Alloza et al., 2013; Füger et al., 2017). Additionally, the high spatial resolution and limited penetration of the MPM technique precludes whole brain imaging, even in mice. Apart from that, significant training and expertise to successfully perform the surgeries, with limited trauma, and the ability to image and reimage the brains of mice requires a significant investment.

Whereas microglia can be classically identified by post-mortem histological methods, histological studies only provide a snapshot in time, obscuring potentially important dynamic processes (Koshinaga et al., 2000; Petersen and Dailey, 2004). Non-invasive neuroimaging techniques such as MRI and PET, with direct clinical applications (Donat et al., 2017), have also faced some technical difficulties and limitations, apart from those inherent to the techniques that were previously discussed. *Ex vivo* histological-MRI approaches have reported hypointensities corresponding to iron deposits, largely associated with activated microglia (Ali et al., 2015; Bulk et al., 2018), however, to our knowledge no *in vivo* studies have been reported, supporting the difficulty to directly assess *in vivo* microglia. PET approaches have included radiolabeled ketoprofen, a selective cyclooxygenase-1 inhibitor, associated with Aβ deposits in animal models (Shukuri et al., 2016) although with limited binding affinity and specificity in human studies (Ohnishi et al., 2016). Different efforts have also been directed to label TSPO, which becomes over-expressed upon activation of microglial cells, revealing increased inflammation that overlaps with Aβ deposition in mild cognitive impairment patients (Parbo et al., 2017). However, translocator protein radioligands present some limitations related to affinity and patients need to be classified as high, mixed and low affinity binders (Hamelin et al., 2016; Knezevic and Mizrahi, 2018). Also, PET does not permit the visualization of microglia at the molecular and cellular levels, nor the ability to obtain the precise timing of their dynamic changes (Kozai et al., 2012), making it hard to detect whether the neuroinflammatory progress occurs early on or later during disease, which is a primary aim of the study of microglia *in vivo*.

An additional challenge in intravital optical imaging is compensating for motion artifacts, particularly with small processes of individual cells, and particularly in awake behaving animals. Even under anesthesia, tissue motion may significantly impair imaging acquisition and resolution. The two major sources of physiological movements are the respiratory and the heart cycles (Vinegoni et al., 2014). Also, common anesthetics may interfere with brain hemodynamics and cellular activity. Therefore, new approaches for imaging the brain in unanesthetized, awake mice, with head fixed systems have been steadily improving (Kuchibhotla et al., 2008; Kuhn et al., 2008). Despite these advances, motion artifacts may need to be further addressed afterwards by image processing (Greenberg and Kerr, 2009; Vinegoni et al., 2014).

In summary, *in vivo* multiphoton imaging is a powerful approach to assess the role of microglia in AD. It allows structural and functional imaging of the living brain, with subcellular resolution, over time. Future studies exploiting this technique should be able to clearly delineate the normal and pathophysiological role of neuroinflammation in the brain, increasing our understanding of the cellular and molecular changes during progression of disease.

## Ethics Statement

All studies were conducted with approved protocols from the Massachusetts General Hospital Animal Care and Use Committee, and in compliance with NIH guidelines for the use of experimental animals or approved by the Animal Care and Use Committee of the University of Cadiz, in accordance with the Guidelines for Care and Use of experimental animals (European Commission Directive 2010/63/UE and Spanish Royal Decree53/2013).

## Author Contributions

CH-B drafted part of the manuscript and reviewed it. BB and MG-A designed, drafted and reviewed the manuscript. All authors contributed to and have approved the final manuscript.

## Conflict of Interest Statement

The authors declare that the research was conducted in the absence of any commercial or financial relationships that could be construed as a potential conflict of interest.
